# ﻿An illustrated key to European genera of Microgastrinae parasitoid wasps (Hymenoptera, Braconidae) with a recent historical and biological perspective and a guide to available species-level identification resources

**DOI:** 10.3897/zookeys.1212.126155

**Published:** 2024-09-23

**Authors:** Mark R. Shaw, Amelie Höcherl, Jose Fernandez-Triana

**Affiliations:** 1 National Museums of Scotland, Edinburgh, UK National Museums of Scotland Edinburgh United Kingdom; 2 SNSB-Zoologische Staatssammlung München, München, Germany SNSB-Zoologische Staatssammlung München München Germany; 3 Canadian National Collection of Insects, Arachnids and Nematodes, Ottawa, Canada Canadian National Collection of Insects, Arachnids and Nematodes Ottawa Canada

**Keywords:** Biology, Europe, identification key, morphology, taxonomy

## Abstract

An illustrated key is provided for the identification of the 20 genera of Microgastrinae so far known to occur in Europe. A brief review of 20^th^ century progress on the group is given. Morphological terms are explained and illustrated, with special reference to the different systems of wing venation employed by past workers on Microgastrinae in Europe, and recommendations are made for future work. For each genus, an outline of species richness, host usage, developmental biology, and particular morphological features is given, and some species that may be difficult to place are highlighted. Available keys for species identification within genera are referenced, with comments, and attention is drawn to species more recently recorded or described up to January 2024. The value of accurate rearing data for parasitoids is emphasised. The references discussed provide a comprehensive overview of the most useful literature for future morphological work on European Microgastrinae. *Glyptapantelesmoldavicus* (Tobias), **comb. nov.** is proposed.

## ﻿Introduction

Microgastrinae is probably the most speciose subfamily in Braconidae (Hymenoptera), with more than 3,000 described species, and the actual total estimated to be up to 40–50,000 species (Fernandez-Triana et al. 2020). They exclusively parasitise all but the more primitive Lepidoptera (i.e., they are parasitoids of Heteroneura, though not Nepticuloidea) and include some important and commonly used parasitoids in biological control efforts against agricultural and forestry pest worldwide (e.g., Whitfield 1997).

The species of Microgastrinae are currently placed in 82 described genera, with the highest number found in tropical regions. The Palaearctic region has the lowest diversity with 28 genera, but even that total includes some Oriental genera that have a few species just entering the southernmost areas of the Eastern Palearctic (Fernandez-Triana et al. 2020). There are just 20 genera of Microgastrinae currently known from Europe. Based on Fernandez-Triana et al. (2020) and papers on the European fauna published after that (Shaw 2020, 2022, 2023a; Shaw and Fernandez-Triana 2020; Shaw and Colom 2023; Höcherl et al. 2024) there are 509 described species recorded in the region, with eight of those being considered as species inquirendae (with uncertain generic placement). The four most speciose genera in Europe are *Dolichogenidea* (109 species), *Cotesia* (106), *Microplitis* (62), and *Microgaster* (54); altogether they represent 65% of all known species in the region. The genera *Glyptapanteles* and *Apanteles* which are the two most speciose at a global scale (Fernandez-Triana et al. 2020) are much less diverse in Europe, with 36 and 30 species, respectively.

There has never been a comprehensive taxonomic key to all genera of European microgastrines, although two papers (Nixon 1965; Mason 1981) dealing with the internal classification of the subfamily on a world basis at least indirectly provided that information. Between 1972 and 1976, Gilbert Nixon (Commonwealth Institute of Entomology, based at the Natural History Museum, London, UK) published, in several papers, an overall revision of the north-western European species of the traditional *Apanteles* (sensu lato) (i.e., species with the fore wing second submarginal cell open distally), and he included a key to the relevant species groups he defined (largely in Nixon 1965) in the second paper of that series (Nixon 1973). A similar approach, derived from Nixon’s work but involving many additional species, was taken by Jenö Papp (Hungarian Natural History Museum, Budapest, Hungary) who published, between 1976 and 1990, a series of papers covering the same concept of *Apanteles* (sensu lato) in the whole of Europe, including a key to (slightly different, often further divided) species groups in his first paper (Papp 1976a). Because many of these species groups of *Apanteles* (sensu lato) were later elevated to genera by Mason (1981) and subsequent authors, the keys of Nixon (1973) and Papp (1976a) can partially work to separate some European genera. Papp (1988) eventually proposed a listing of the European species of the former *Apanteles* (sensu lato) under the later generic concepts of Mason (1981). However, many species have since been assigned to different genera (cf. Fernandez-Triana et al. 2020) and, in any case, none of the genera with a complete second submarginal cell (= areolet) were included in *Apanteles* and thus they were not covered by Nixon (1973) [but see Nixon (1965) for further elucidation of the world genera of Microgastrinae considered at the time, which is now badly outdated]. The literature listed here gives details of these many papers, which are cited in the entries for each genus following the key.

It is widely recognised that the current generic classification of Microgastrinae as a whole is imperfect. Ongoing and future phylogenetic studies might eventually radically overturn the present generic concepts, but a more durable and well-supported classification is not likely to be achieved quickly. This paper follows the generic concepts of Microgastrinae as discussed in Fernandez-Triana et al. (2020). However, even within the European fauna, some generic boundaries are poorly defined. That is especially the case between *Dolichogenidea* and both *Apanteles* and *Pholetesor* and, occasionally, between *Protapanteles* and both *Glyptapanteles* and *Cotesia*, problems recognised long ago by van Achterberg (2003) although his solution was widely rejected.

The importance that reared material has had in the study of European Microgastrinae is impossible to overstate. Nixon’s revisions, in particular, benefited enormously from huge rearing efforts undertaken in Britain by Richard Ford on behalf of Douglas Wilkinson, whose pioneering work on *Apanteles* (sensu lato) (e.g., Wilkinson 1945) was curtailed by his death in action during the Second World War. Wilkinson’s work, which had a strong focus on interpreting historical names, was subsequently picked up by Nixon (accessing also the vital reared specimen base), albeit with a different approach. Nixon’s great skill was to delimit the material before him to species, but he was less concerned with pre-existing names and, if no existing name was easily found, he took the pragmatic solution and described species as new. His keys include supplementary entries and pay invaluable attention to the host repertoires of the species he treats, so his work is especially helpful in anchoring biological information to a name. Papp’s keys have no annotations and pay practically no regard at all to hosts. He did, however, make greater effort to take account of existing names and he proposed much synonymy although, as older types were often no longer in existence, some uncertainty was introduced (he did not designate neotypes). Van Achterberg’s (1997) revision of the Haliday collection, rich in type material, did much to clarify the names of relevant microgastrine species (but see Shaw 2003, 2012). DNA barcoding is now increasingly providing new perspectives on the species-level taxonomy, and large barcode libraries are being built.

Reared specimens with accurate host data continue to be tremendously valuable and will inevitably have continued importance as taxonomic knowledge evolves with new molecular techniques (e.g., Höcherl et al. 2024). For many species, no host is reliably known and, even for those with clear host data at one time of year, it is often the case that an additional, still undiscovered, host is needed to complete the annual cycle. Most unfortunately, owing to errors of various kinds in the published literature, sources such as Taxapad (Yu et al. 2012, 2016) give a very distorted and untrustworthy view of host relations resulting from unfiltered compilations of literature records (Shaw 1994). In the present context, this also applies to the abstracted host lists given by Papp (1988, 1990) in the concluding parts of his work on *Apanteles* (sensu lato). In reality, at a species level the great majority of Microgastrinae are, to a greater or lesser degree, very host-specialised.

Some Microgastrinae make highly distinctive cocoons (or cocoon masses); dried cocoons, and host remains, should always be preserved with reared specimens, preferably in a gelatine capsule pinned with the adult(s). Taking reference photos of cocoons in situ can also be useful. Cocoons of gregarious broods should be kept together, not split up even if (preferably) the adults are individually mounted. Some general advice on rearing and preserving parasitoids is given by Shaw (1997). Unambiguous and clear labelling of reared specimens is of paramount importance, and it is vital that any doubt as to the host’s identity is clearly expressed (Shaw 2023b).

Here, we present the first comprehensive and illustrated key to the genera of Microgastrinae known from Europe according to present views of generic boundaries. For each included genus we also provide an outline of species-richness in Europe (based on Fernandez-Triana et al. 2020 but updated to January 2024), a brief indication of host relations and developmental biology, and comment on the most appropriate and available literature for species-level determination.

## ﻿Methods and terminology

We consider Europe here as defined by Fauna Europaea (De Jong et al. 2014), which includes the British Isles, European mainland plus the Macaronesian islands (excluding Cape Verde Islands), Franz Josef Land, Novaya Zemlya and Cyprus; but excludes Turkey, most of the territory between the Black and Caspian Seas, and northern Africa. The geographic boundaries include East: Ural (60°E), West: Atlantic Ocean (Mid-Atlantic Ridge) (30°W), South: Mediterranean (35°N), North: Arctic Islands (82°N).

This paper follows the generic concepts of Microgastrinae as discussed in Fernandez-Triana et al. (2020). As indicated above, the generic placement of some species is currently somewhat unclear; we assign species according to the morphological traits they exhibit (i.e., characters as interpreted and used in this key), although we can expect that in the future generic placements of some may be revised.

Morphological terms used in the key below mostly follow Huber and Sharkey (1993), Whitfield (1997), Karlsson and Ronquist (2012), and Fernandez-Triana et al. (2014), which are usually included in the Hymenoptera Anatomy and Ontology (HAO) website (http://portal.hymao.org/projects/32/public/ontology/). However, most of the published literature on European and Palaearctic Microgastrinae has followed morphological terms sensu Nixon, Papp, or van Achterberg. The terminologies are particularly different for wing venation. Therefore, we illustrate below (Figs 22–25) four different venation systems (sensu, respectively, Nixon 1965, Papp 1976a, van Achterberg 1979, and Sharkey and Wharton 1997) to facilitate the understanding and use of historical literature. The drawing is schematic, depicting a typical wing of *Dolichogenidea* sp., the enlarged inset shows in detail the wing venation if a complete areolet is present; in that case the vein and cell names may differ slightly. In this paper we are following Sharkey and Wharton (1997), therefore Fig. 25 below should be used as the main reference for wing venation.

Other morphological terms are also different, but easier to relate; examples include (HAO term first, other uses second) anteromesoscutum vs mesoscutum, metacoxa vs hind coxa, first segment of tarsus vs basitarsus, pterostigma vs stigma. One specific term, widely used in European papers on Microgastrinae, is the “basal field” (of the second tergite), usually referring to what HAO and American authors name “mediotergite 2” or at times “tergum 2” (when a median area is not differentiated from the rest of the tergum). We refer to it here as T2. The morphological characters (other than wing veins) most used in the key are illustrated below in Fig. 26.

By the informal term ‘macrolepidoptera’ we mean especially the superfamilies Papilionoidea, Drepanoidea, Lasiocampoidea, Bombycoidea, Geometroidea, and Noctuoidea, most of whose larvae lead at least partly exposed lives, but we would also include more basal groups such as Zygaenoidea with similar feeding ecology. The term ‘microlepidoptera’ covers the remainder, generally smaller species with more concealed larval feeding habits.

Specimens (females, unless indicated otherwise) were photographed using a Keyence VHX-6000 or VHX-1000 digital microscope and z-stacks were computed using the built-in software of the microscope. Subsequent processing and construction of image plates and figures were effected using Photoshop and Inkscape. Because the photos are stacked, allowance needs to be made for some distortion of dimensions when the elements illustrated were not co-planar.

## ﻿Results

### ﻿Key to the European genera of Microgastrinae

This key works best for female specimens. The European fauna of many genera is much less morphologically diverse than the world fauna, so it is important to note that this key will only work well in the European context (and even there, moderately for some taxa). Some outlying species are nevertheless difficult to key and are mentioned in the relevant couplets, and some genera come out in more than one place. For morphology see the standards detailed in Methods (especially Figs 25, 26).

**Table:** 

1	Fore wing with second submarginal cell (= areolet) entirely to almost entirely delimited by pigmented veins (rarely with postero-lateral sides of cell absent due to transparent or spectral veins, but overall outline of areolet still clearly marked) (Fig. 1A–D), rarely indistinct owing to adpression (closeness) of veins (*Paroplitis*) or areolet minute and very proximal (*Choerasgielisi*)	**2**
–	Fore wing clearly without second submarginal cell, i.e., no veins at postero-lateral sides to delimit an areolet, at most with stub of 3RS present (Fig. 1A'–D')	**8**
2(1)	Metacoxa ~ 2.0 × as large as mesocoxa and < 0.3 × entire metasoma length (Fig. 2A); posterior end of metacoxa not surpassing posterior margin of T2 (usually not surpassing posterior margin of T1) (Fig. 2B); metatibial spurs of similar length and shorter than 0.5 × length of first segment of metatarsus (Fig. 2C)	***Microplitis***
–	Metacoxa ~ 3.0 × as large as mesocoxa and at least 0.3 × metasoma length (usually more) (Fig. 2A'); posterior end of metacoxa surpassing posterior margin of T2 (Fig. 2B'); inner metatibial spur often longer than outer spur and usually > 0.5 × length of first segment of metatarsus (Fig. 2C')	**3**
3(2)	T1 significantly widest at posterior margin (Fig. 3A, B) and without median longitudinal sulcus (although it may be circularly excavate in anterior half (Fig. 3A)); T2 broad, rectangular to sub-rectangular; T1 and T2 strongly sculptured (Fig. 3A, B), only rarely with T2 smooth (*Microgasterpolitus* Marshall and largely also *M.nobilis* Reinhard); setose part of ovipositor sheath often extending well beyond hypopygium, **if** by not much more than length of second segment of metatarsus (*M.raschkiellae* Shaw), **then** T2 virtually rectangular, ~ 3.0 × wider than long, and evenly rugose	**4**
–	T1 shape variable (Fig. 3A', B') but rarely wider at posterior margin (**if** so, **then** with strong medial longitudinal sulcus (Fig. 3B')); T2 shape variable but very rarely broad or rectangular; T1 and T2 variously sculptured, but often not strongly so; extension of ovipositor in relation to hypopygium variable	**5**
4(3)	Flagellomeres with placodes arranged irregularly (often in three ranks but sometimes ranks not clearly defined), thus proximal and middle flagellomeres not appearing subdivided in two (Fig. 4A); apical (fifth) segment of all tarsi enlarged, usually longer than combined length of third and fourth segments (Fig. 4B); metatarsal claws large and simple (without lobe or spines); in lateral view, mesosoma elongate so body usually looking slightly depressed (Fig. 4C)	***Hygroplitis***
–	Flagellomeres with placodes arranged in two ranks, thus proximal and middle flagellomeres appearing subdivided in two (Fig. 4A'); apical (fifth) segment of all tarsi of normal size, and usually not longer than combined length of third and fourth segments (Fig. 4B'); metatarsal claws often with one to several spines, rarely large and simple (*Microgasterauriculata* (Fabricius), *M.deceptor* Nixon and *M.stictica* Ruthe) or with a basal lobe (*M.deductor* Nixon); in lateral view, body not depressed (Fig. 4C')	***Microgaster***
5(3)	Hypopygium ventrally desclerotised and with several pleats (Fig. 5A); setose part of ovipositor sheaths > 0.5 × metatibia length (Fig. 5A); ovipositor sheaths densely covered by setae on all or most of its length (Fig. 5A); fore wing areolet **either** very small (outer side very proximal and sometimes hard to see except by vein thickening) **or** very often partially delimited (posterolateral sides weakly marked by transparent veins or just by denser setae)	***Choeras*** (in part)
–	Hypopygium fully sclerotised or, at most, with small fold ventrally setting off a more translucent area but without pleats (Fig. 5A'); projecting part of ovipositor sheaths shorter than 0.5 × metatibia length, often much shorter (Fig. 5A'); ovipositor sheaths usually with few, sparser setae which are mostly on apical tip (except *Paroplitis*) **or** with setae not visible; fore wing with areolet often large and clearly delimited by pigmented veins, but sometimes areolet with outer side mostly spectral, and occasionally marked only by thickening of vein 3RS and/or vein r-m	**6**
6(5)	Hypopygium slightly folded along middle line ventrally, setting off a more translucent and flexible area; setose part of ovipositor sheath projecting ~ 0.4 × length of metatibia (Fig. 5A'); female antenna shorter than body (Fig. 6A), with most flagellomeres with single rank of placodes (thus proximal and central flagellomeres not appearing subdivided); legs short and robust, especially metafemur (Fig. 6A); metasomal terga mostly smooth; relatively small body size, < 2.5 mm	***Paroplitis***
–	Hypopygium fully sclerotised, ventrally without any pleats (Fig. 6A'); ovipositor sheaths projecting scarcely more than length of first segment of metatarsus (often less) and **either** usually with few setae which are mostly towards apex (Fig. 7A) **or** with not or scarcely visible setae (Fig. 7A'); female antenna often (but not in *Diolcogasterspreta* (Marshall)) longer than body and with proximal and middle flagellomeres with two ranks of placodes (thus flagellomeres appearing subdivided in two); legs variable, but rarely short and robust (*D.spreta*); metasomal terga variable, but usually T1 and T2 sculptured (often strongly) (Fig. 7B, B'); body size variable but often > 2.5 mm	**7**
7(6)	Ovipositor sheaths with setae apically, often with one or a few setae that are thicker and larger than the rest (Fig. 7A); T1 usually with strong (but sometimes weak) median longitudinal groove over most of its length (Fig. 7B); posterior band of scutellum with sculpture medially so that scutellum is rugose or rarely punctate adjacent to metanotum centrally (Fig. 7C) (but *Diolcogasterflavipes* (Haliday) without sculpture)	***Diolcogaster***
–	Ovipositor sheaths without setae (or with few and extremely small setae) (Fig. 7A'), though setae conspicuous in *R.desueta*; T1 with broad depression antero-medially, without median longitudinal groove (Fig. 7B'); scutellum usually with posterior band lacking rugosity medially, leaving a smooth area centrally between it and metanotum (Fig. 7C') (but hardly so in *R.marginata*)	***Rasivalva***
8(1)	Hypopygium ventrally desclerotised, with several pleats (Fig. 8A) **or** slightly folded along middle line ventrally, setting off a more translucent and flexible area (Fig. 8B); ovipositor sheaths often > 0.5 × metatibia length (Fig. 8A); ovipositor sheaths with dense and conspicuous setae on all or most of its exposed length (Fig. 8A, B)	**9**
–	Hypopygium fully sclerotised, ventrally without any pleats (Fig. 8A', B') (but in some dead specimens with shrunken metasoma it might look as if weakly/variably folded); ovipositor sheaths usually projecting beyond hypopygium < 0.5 × metatibia length, often much shorter (Fig. 8A', B'), but sometimes sheaths 0.5 × (and then practically straight) or rarely 1.0 × (and then strongly downcurved) and in both these cases projecting part is setose (Fig. 16A–C); otherwise ovipositor sheaths **either** usually with few, sparser setae (Figs 16A'–C', 17A', B') or setae mostly towards apical tip **or** rarely setae not visible (Fig. 17A, B)	**16**
9(8)	Hypopygium mostly sclerotised but slightly folded along the middle line setting off a more translucent and flexible area (Figs 8B, 9A, B, 10B, B')	**10**
–	Hypopygium desclerotised (membranous) ventrally, with multiple expandable pleats (Figs 8A, 9A', B')	**11**
10(9)	Tarsal claws pectinate (Fig. 10D); flagellum very bristly (Fig. 10A); setose part of ovipositor sheath 0.6–0.7 × metatibia length (Fig. 10B); T1 broad, widely rounded posteriorly (Fig. 10C)	***Choeras*** (in part)
–	Tarsal claws simple; flagellum not exceptionally bristly (Fig. 10A'); ovipositor sheath < 0.6 × metatibia length (usually < 0.5 ×); T1 often more or less posteriorly narrowing or truncate (Fig. 10C', D') or approximately quadrate in *Pholetesormaritimus* (Wilkinson)	***Pholetesor***
11(9)	Wing membrane dark brown; fore wing vein R1 clearly shorter than pterostigma; legs, including metatibial spurs, black (Fig. 11)	***Napamus***
–	Wing membrane usually hyaline (**if** exceptionally strongly infumated and legs dark (*Dolichogenideagagates* (Nixon)) **then** metatibial spurs much paler than metafemur); fore wing vein R1 usually longer than or subequal to pterostigma (rarely shorter)	**12**
12(11)	Propodeum uniformly rugose, without any carinae particularly marked (Fig. 12A); scutellum usually with posteromedian band centrally rugose (abutting metanotum) (Fig. 12A); inner margins of eyes usually convergent below (Fig. 12B); fore wing vein R1 shorter than pterostigma (Fig. 12C); usually T3–T6 with medioapical weakly sclerotised area, tergites appearing pushed forward medially (Fig. 12D)	***Illidops***
–	Propodeum **either** mostly smooth, with or without medial longitudinal carina (Figs 13A, 14A, A'), **or** fully or partially areolated (Fig. 14C', D') **or** with a few rugae from posterior margin before its area of articulation of metasoma (= nucha, Fig. 14B'); scutellum without posteromedian rugosity (so that a smooth area abuts metanotum centrally as in Fig. 13B); inner margins of eyes usually not or only slightly convergent below; fore wing vein R1 usually subequal to or longer than pterostigma (rarely shorter); T3–T6 usually without medioapical weakly sclerotised area, tergites in most cases not appearing pushed forward medially (there are several exceptions in *Apanteles* and *Dolichogenidea*)	**13**
13(12)	Propodeum mostly smooth but with median longitudinal carina, usually strongly defined (Fig. 13A) and raised (sometimes weak or interrupted); lateral face of scutellum with the polished area (= lunula) 0.7 × or more height of lateral face (Fig. 13B) (so that sculptured area between lateral and dorsal faces is narrow); fore wing vein r usually much longer than the short 2RS which it meets in a curve with a stub of 3RS at most weakly indicated (Fig. 13C); hind wing usually with vein cu-a sinuous or thickened at posterior end	***Iconella***
–	Propodeum **either** mostly smooth (Fig. 14A', B') **or** fully or largely areolated (Fig. 14C', D'), **if** with median longitudinal carina then only weakly indicated as longitudinal sculpture marking origin of radiating vermiculate rugulosity (Fig. 14A) **and** fore wing vein r straight and not greatly longer than the long and straight vein 2RS, which it meets at an acute angle with stub of 3RS prominent (Fig. 14B); lateral face of scutellum variable but usually with polished area < 0.7 × height of lateral face (as in Fig. 12A); hind wing with vein cu-a usually weakly incurved but not sinuous	**14**
14(13)	Propodeum with vermiculate rugulosities radiating outwards from a central line of more longitudinal sculpture at least suggestive of a keel (Fig. 14A) **and** fore wing r straight and not or scarcely longer than long and straight 2RS (Fig. 14B), which it meets at an acute angle with stub of 3RS prominent; T1 at least weakly (often strongly) wedge-shaped (Fig. 14C, D)	***Choeras*** (in part)
–	Propodeum **either** mostly smooth (sometimes with a few rugae on posterior margin, Fig. 14A', B'), **or** with carinae marking a complete or partial areola (Fig. 14C', D'); fore wing venation variable but usually not as above; T1 shape variable but rarely wedge-shaped	**15**
15(14)	Hind wing with vannal lobe from slightly concave (Fig. 15B) to straight (Fig. 15A), medially without setae (**if** rarely with some setae **then** setae very sparse and very short) (Fig. 15A, B); **if** anteromesoscutum with distinct punctation **then** punctures near posterior margin elongate through fusion with adjacent ones (Fig. 15C, D)	***Apanteles***
–	Hind wing with vannal lobe evenly convex (Fig. 15A'), rarely vannal lobe with almost straight margin (Fig. 15B'), lobe medially with fringe of comparatively clear setae, which are uniformly dense (Fig. 15A') (rarely lobe with almost straight margin and few small setae or no setae, e.g., *Dolichogenideasicaria* (Marshall) Fig. 15B'); **if** anteromesoscutum with distinct punctation **then** punctures near posterior margin not fusing with adjacent ones (Fig. 15C', D')	***Dolichogenidea***
16(8)	Ovipositor sheaths setose over most of their length, comparatively long, **either** extending beyond hypopygium by ~ 0.5 × metatibia length and nearly straight, **or** approximately as long as metatibia and strongly downcurved (Fig. 16A–C)	***Sathon***
–	Ovipositor sheaths without evident setae (Fig. 17A, B) or with fewer setae or setae apically and subapically (Figs 16A'–C', 17A', B'), sheath extending beyond hypopygium by < 0.5 × metatibia length, usually much shorter (Fig. 16A', C'), **if** rarely ~ 0.5 × metatibia length, **then** dagger-shaped and almost glabrous (*Glyptapantelesliparidis* (Bouché), Fig. 16B') **or** propodeum with strong carination pattern including median carina (a few *Cotesia* such as *C.hyphantriae* (Riley, 1887))	**17**
17(16)	Ovipositor sheaths without setae or, at most, with very few and very small setae apically, almost invisible (Fig. 17A, B)	**18**
–	Ovipositor sheaths with some visible setae at least apically and often subapically (Fig. 17A', B') (almost glabrous in *Glyptapantelesliparidis*, but then sheaths longer, ~ 0.5 × metatibia, Fig. 16B')	**19**
18(17)	Antenna approximately same length as body or slightly longer; proximal and middle flagellomeres with placodes arranged in two rows and more or less appearing divided; legs not particularly short or stout; body size comparatively larger, usually > 3.0 mm (Fig. 18A)	***Distatrix***
–	Antenna much shorter than body; flagellomeres largely with single rank of placodes; legs, especially femora, short, stout and flattened; body size comparatively smaller, < 2.5 mm (Fig. 18A')	***Venanides***
19(17)	Propodeum with strongly defined median longitudinal carina standing out from mostly smooth and shiny background (in *D.carbonaria* (Wesmael) propodeum with some sculpture centrally, Fig. 19C) **and** T2 more or less rectangular in shape and of comparable length to T3 **and** at least posterior half of T1 and most of T2 sculptured (Fig. 19A–C); lateral margin between T2 and T3 appearing slightly indented in dorsal view as T3 posterior margin is slightly wider than T2 posterior margin (Fig. 19A–C); apical segment of protarsus simple	***Deuterixys***
–	Propodeum variably sculptured (Figs 20A–E, 21A–E, A'–E'), **if** rarely with median longitudinal carina standing out from weak surrounding sculpture **then** T2 trapezoidal or subtriangular in shape and shorter than T3 **and/or** most of T1 and T2 weakly or not sculptured; lateral margin between T2 and T3 not appearing slightly indented in dorsal view because T3 posterior margin is not wider than T2 posterior margin (in few cases T3 posterior margin is wider but then T2 has a non-rectangular shape); apical segment of protarsus often simple but in each of the following three genera there are species in which it bears a more or less strong and curved spine	**20**
20(19)	Propodeum usually rugose, often with more or less clear carination, including median longitudinal carina (which might be partially obscured by surrounding strong sculpture, or rarely absent), and often (though less noticeably) a partial to almost complete transverse carina (Fig. 20A–E); T1 usually more or less rugose and considerably < 2.0 × longer than wide, never evenly narrowing posteriorly (usually slightly widening or sometimes parallel-sided and only slightly rounded or narrowed at extreme posterior); T2 usually at least largely rugose (but rarely smooth) and rectangular or subquadrate (though often with anteriorly converging lateral sulci marking off a trapezoidal area), **if** appearing triangular **then** wider than long (T2 largely smooth and strongly rounded anteriorly in *Cotesiagades* (Nixon) and *C.glabrata* (Telenga), or triangular in *C.hispanica* (Oltra and Falco)); T2 usually a little shorter but sometimes subequal to T3; T3 sometimes anteriorly rugose (rarely completely, e.g., *Cotesiainducta* (Papp), Fig. 20A)	***Cotesia***
–	Propodeum usually not as sculptured, either completely smooth or with some sculpture mostly in posterior half, transverse carinae always absent, median longitudinal carina usually absent, **if** almost complete (*Glyptapantelespallipes* (Reinhard)) **then** surrounding sculpture relatively weak; T1 often (many *Glyptapanteles*) fully 2.0 × as long as wide, in which case usually rather evenly tapering posteriorly (but *Protapantelesparallelus* (Lyle) has long T1 which is parallel-sided its whole length), **if** (frequently) shorter **then** at least strongly rounded posteriorly (most *Protapanteles*); T2 either more or less triangular or trapezoidal, **if** more or less rectangular **then** anterior margin with rounded corners; T2 shorter than T3; T3 always smooth (Fig. 21A–D, A'–E')	**21**
21(20)	Propodeum usually with some sculpture, especially on posterior half, but never with median carina (Fig. 21A–D); T1 often moderately broad and parallel-sided anteriorly (but more barrel-shaped in *Protapantelespopularis* (Haliday)), broadly rounded posteriorly (narrow and not rounded in *P.parallelus* (Lyle)); T1 and T2 usually with some sculpture, at least matt; T2 usually rectangular with anterior margin rounded but more or less triangular in several species and then sometimes large (e.g., *P.anchisiades* (Nixon) and *P.mandanis* (Nixon)); protarsus in most species with curved spine on apical segment	***Protapanteles***
–	Propodeum usually smooth, but sometimes with almost complete median carina (*Glyptapantelespallipes* (Reinhard)); T1 usually rather slender, **if** not wedge-shaped **then** at least strongly narrowing posteriorly; T1 and T2 often almost smooth and shining; T2 subtriangular or trapezoidal and in one species (*Glyptapantelesmoldavicus* (Tobias)), with a raised longitudinal ridge (Fig. 21E'), sometimes narrow and longer than wide; **if** protarsus with curved spine (present in a few common species) **then** T1 is clearly wedge-shaped, narrowing over most of its length (Fig. 21A'–E')	***Glyptapanteles***

**Figure 1.**
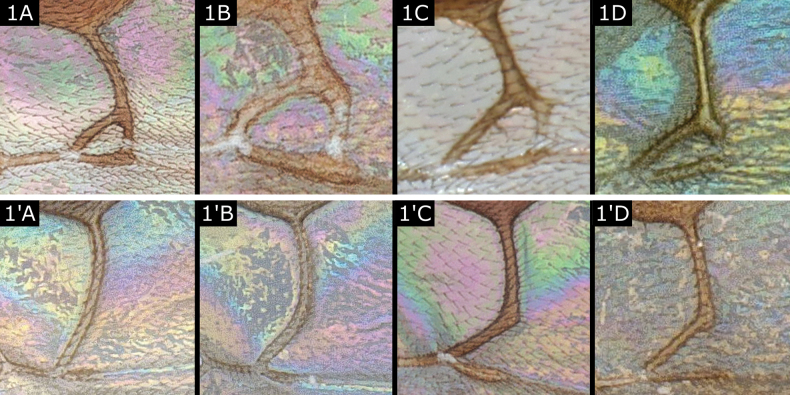
**A***Microgastercaris***B***Microplitiskewleyi***C***Microgasterraschkiellae***D***Diolcogasteralvearia***A**' *Glyptapantelesinclusus***B**' *Dolichogenideaanarsiae***C**' *Glyptapantelespopovi***D**' *Choerasparasitellae*.

**Figure 2.**
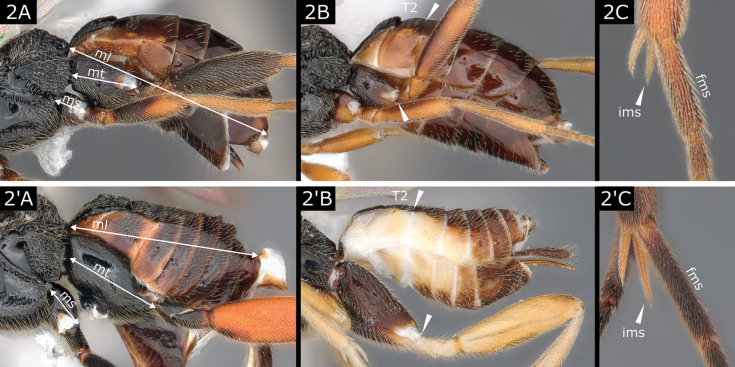
**A, C***Microplitiscoactus***B***Microplitiskewleyi***A**' *Microgasternervosae***B**' *Choerasciscaucasicus***C**' *Microgasterprocera*. Abbreviations: fms- first metatarsus segment; ims- inner metatibial spur; ml metasoma length; ms mesocoxa; mt metacoxa; T2 second tergite.

**Figure 3.**
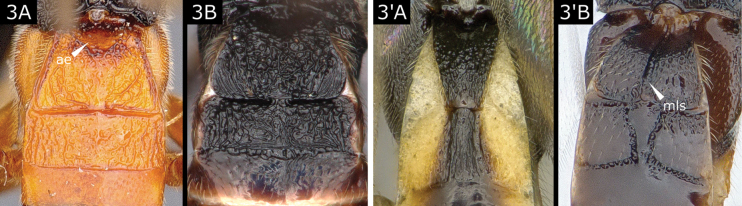
**A***Hygroplitisrussata***B***Microgasterraschkiellae***A**' *Rasivalvamarginata***B**' *Diolcogasterspreta*. Abbreviations: ae anterior excavation; mls medial longitudinal sulcus.

**Figure 4.**
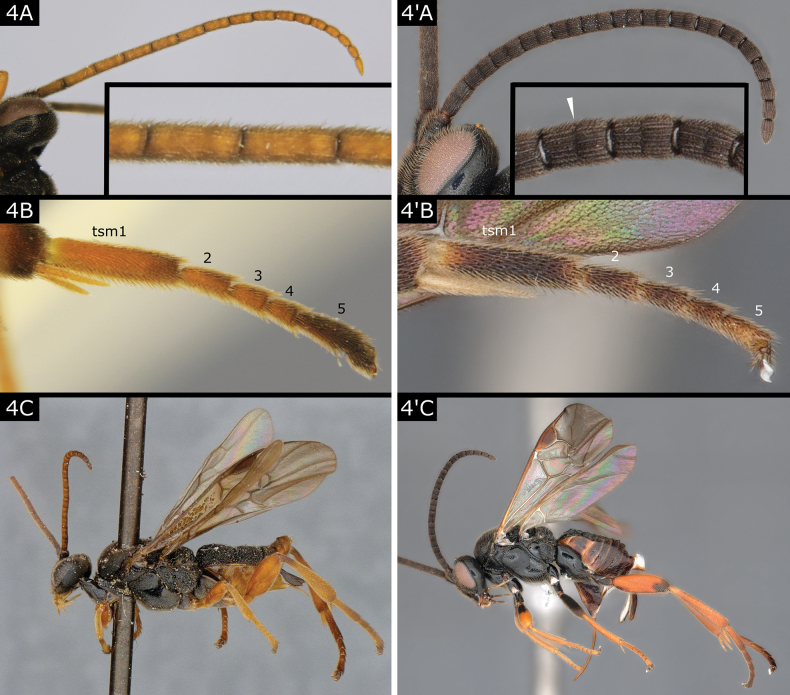
**A, B***Hygroplitisrussata***C***Hygroplitisrugulosa***A**' *Microgasternervosae***B**' *Microgastercaris***C**' *Microgasternervosae*. Abbreviation: tsm tarsomere.

**Figure 5.**
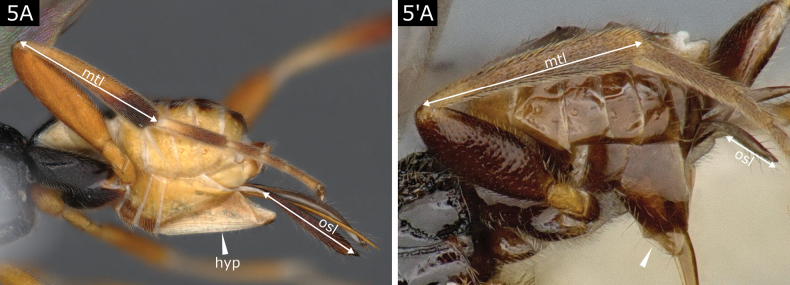
**A**Choerascf.dorsalis**A**' *Paroplitiswesmaeli*. Abbreviations: hyp hypopygium; mtl metatibia length; osl ovipositor sheath length.

**Figure 6.**
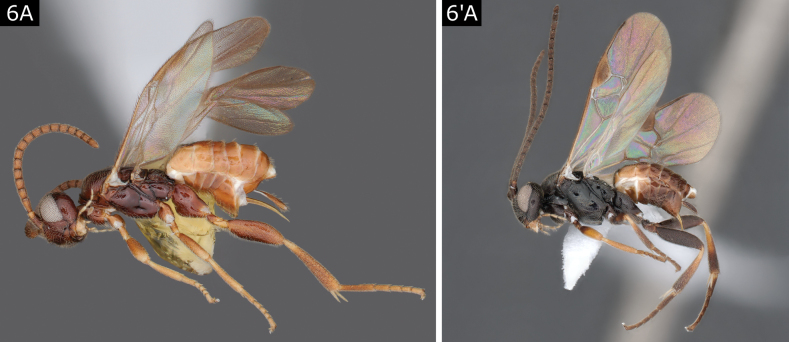
**A***Paroplitiswesmaeli***A**’ *Diolcogasterclaritibia*.

**Figure 7.**
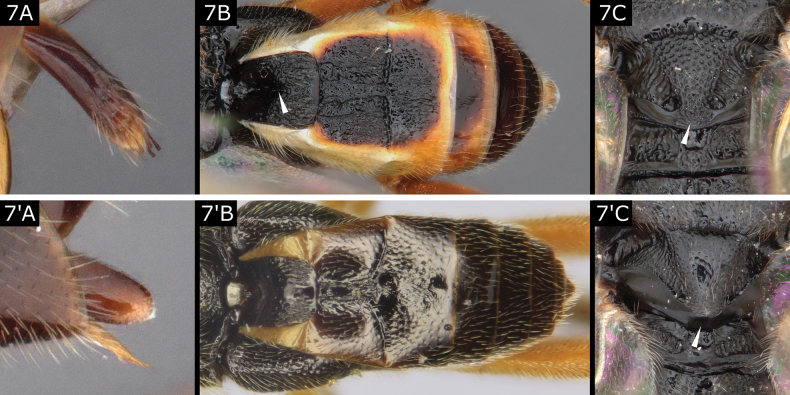
**A–C***Diolcogasterhinzi***A', C**' *Rasivalvacalceata***B**' *Rasivalvacircumvecta*.

**Figure 8.**
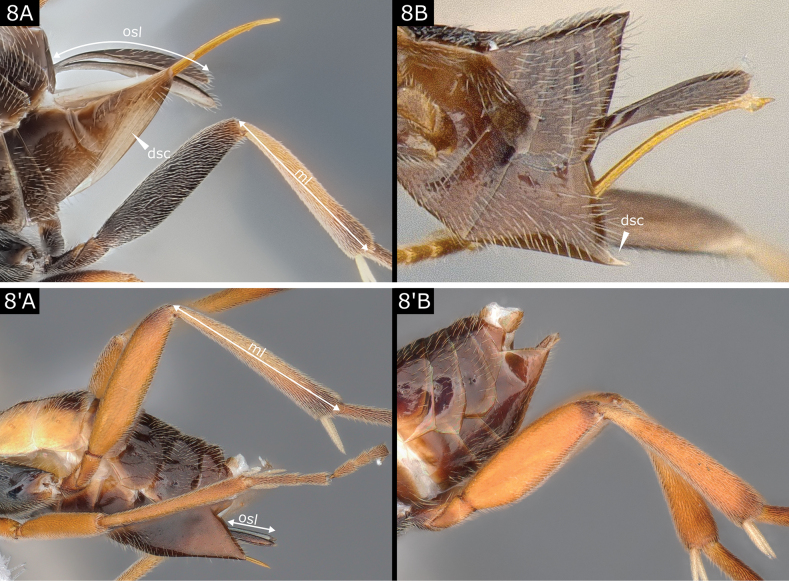
**A***Illidopssplendidus***B***Pholetesorbedelliae***A**' *Glyptapantelespopovi***B**' *Cotesiarisilis*. Abbreviations: ml metatibia length; osl ovipositor sheath length; dsc desclerotised area of hypopygium.

**Figure 9.**
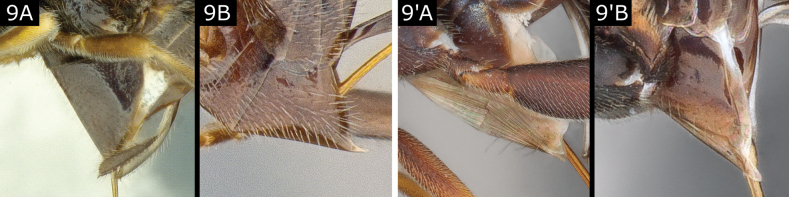
**A***Choerasvalidus***B***Pholetesorbedelliae***A**' *Apantelesgalleriae***B**' *Dolichogenideacheles*.

**Figure 10.**
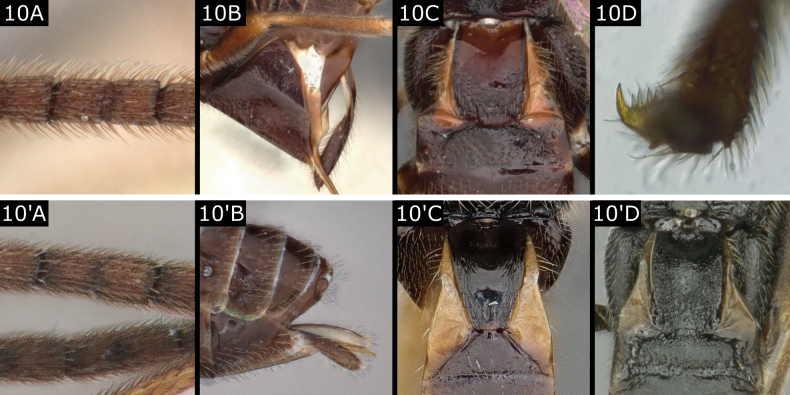
**A–D***Choerasvalidus***A’, B**’ *Pholetesorbedelliae***C**’ *Pholetesorcircumscriptus***D**’ *Pholetesorviminetorum*.

**Figure 11.**
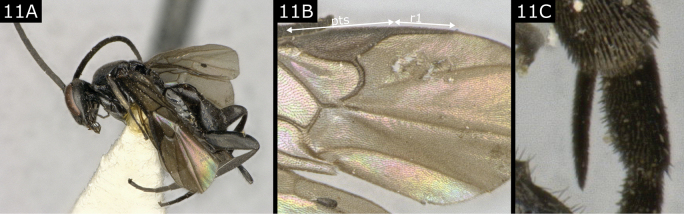
*Napamusvipio*. Abbreviation: pts pterostigma.

**Figure 12.**
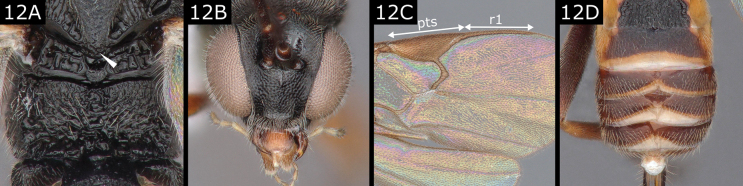
*Illidopsbutalidis*. Abbreviations: pts pterostigma; r1 radial vein.

**Figure 13.**
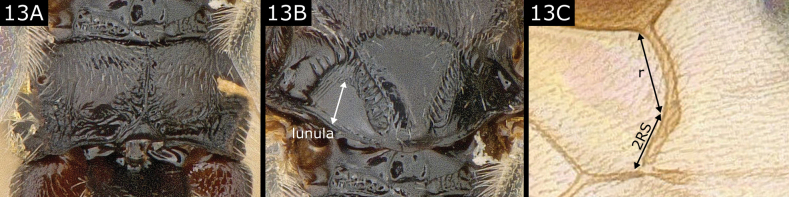
*Iconellamerula*.

**Figure 14.**
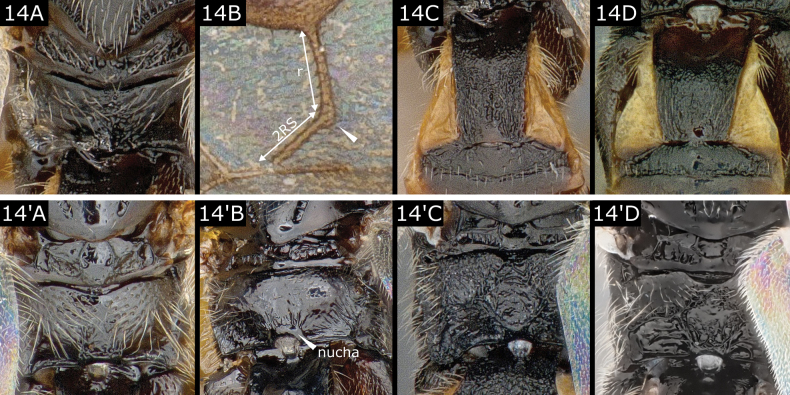
**A–C***Choerasparasitellae***D***Choerasarene***A**' *Dolichogenideabreviventris***B**' *Dolichogenideagracilariae***C**' *Apantelescarpatus***D**' *Dolichogenideacerialis*.

**Figure 15.**
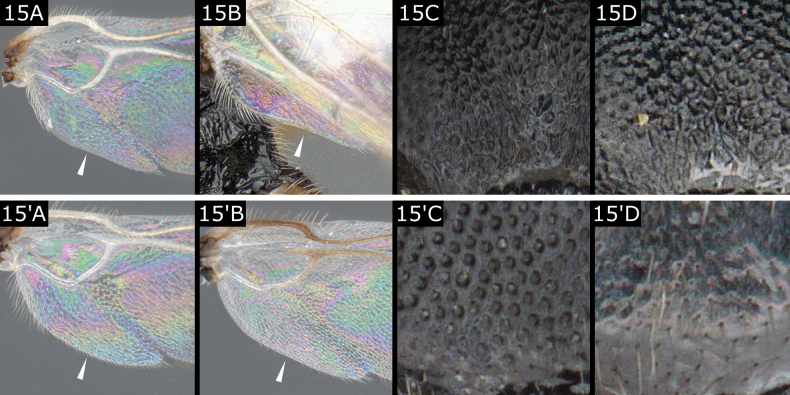
**A, C***Apantelesbrunnistigma***B, D***Apanteleshemara***A**' *Dolichogenideacandidata***B**' *Dolichogenideasicaria***C**' *Dolichogenideacerialis***D**' *Dolichogenideasicaria*.

**Figure 16.**
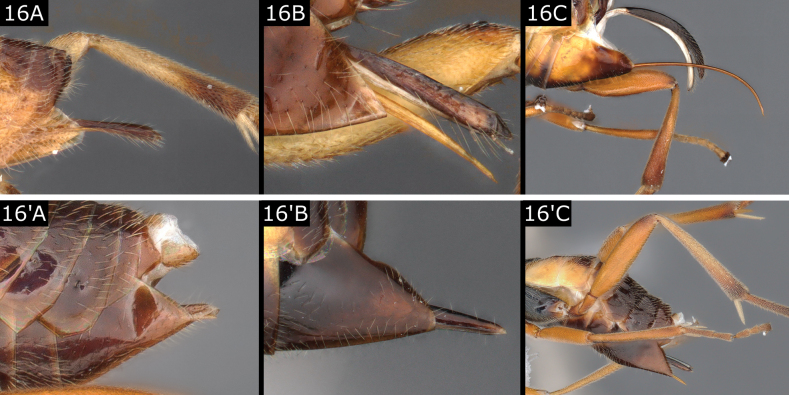
**A***Sathoneugeni***B***Sathonlateralis***C***Sathonfalcatus***A**' *Cotesiarisilis***B**' *Glyptapantelesliparidis***C**' *Glyptapantelespopovi*.

**Figure 17.**
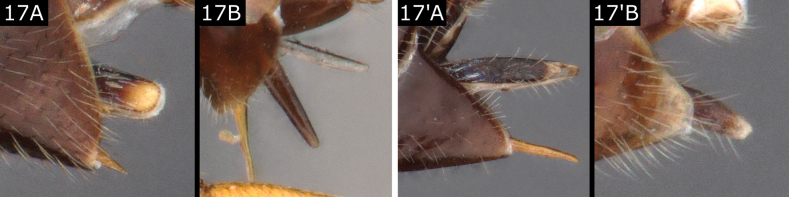
**A***Distatrixformosa***B***Venanidescarcinae***A**' *Protapantelesandromica***B**' *Glyptapantelespallipes*.

**Figure 18.**
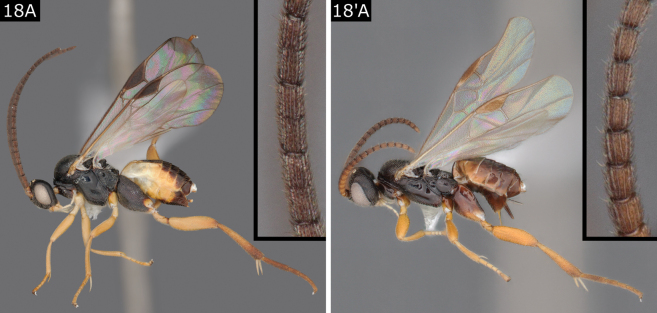
**A***Distatrixformosa***A**' *Venanidescarcinae*.

**Figure 19.**
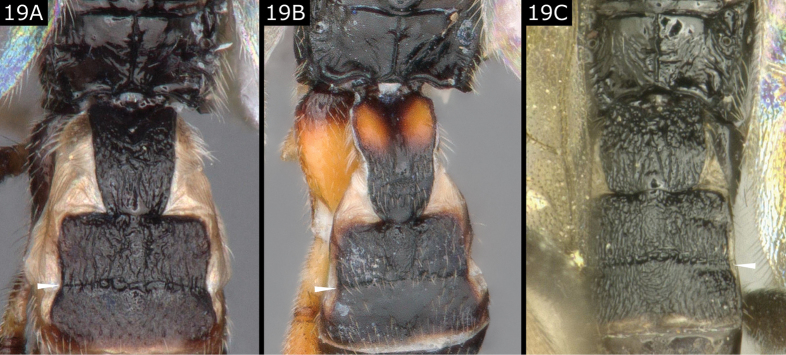
**A***Deuterixysrimulosa***B***Deuterixysplugarui***C***Deuterixyscarbonaria*.

**Figure 20.**
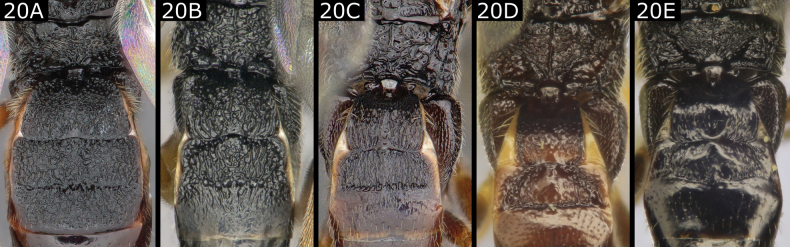
**A***Cotesiainducta***B***Cotesiacallimone***C***Cotesiacajae***D***Cotesiaonaspis***E***Cotesiaglabrata*.

**Figure 21.**
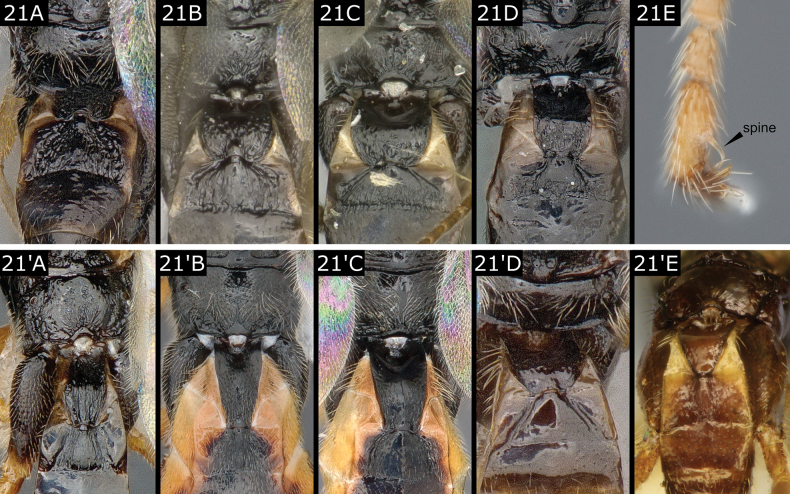
**A***Protapantelesanchisiades***B***Protapantelesenephes***C***Protapantelespopularis***D***Protapanteleshirtariae***E***Protapanteles* sp. apical segment of protarsus **A**' *Glyptapantelesfulvipes***B**' *Glyptapantelespopovi***C**' *Glyptapantelesindiensis***D**' *Glyptapantelesinclusus***E**' *Glyptapantelesmoldavicus* (male).

[Limits between *Cotesia* and *Protapanteles* and especially between *Protapanteles* and *Glyptapanteles* are sometimes very vague, with characters mentioned in the key not always clear-cut; see below for additional comments].

### ﻿Species identification of European Microgastrinae

Many species of European Microgastrinae can be identified (although not always easily: access to an accurately identified reference collection is invaluable) by using the papers of Nixon (1965, 1968, 1970, 1972, 1973, 1974, 1976) and Papp (1973, 1974, 1976a, 1976b, 1978, 1979, 1980, 1981, 1982, 1983, 1984a, 1984b, 1986a, 1986b, 1987, 1988). Nixon’s keys are especially helpful because additional characters, comments, and comparisons are given for each included species. Some works by Papp, particularly when a new species is being described, give tables of characters comparing species; however, the species compared are not always apposite. Two papers from Anatoly G. Kotenko, which covered mostly the area of the former Soviet Union, are also potentially informative, although difficult to use within a European context. Tobias and Kotenko (1986), while being heavily derived from Nixon’s keys, included a large number of species from the eastern Palearctic, but there is not much additional information on species beyond the key characters; Kotenko (2007a, in Russian but with illustrations) focused on species from the Russian Far East, but some European species are also included.

**Figure 22.**
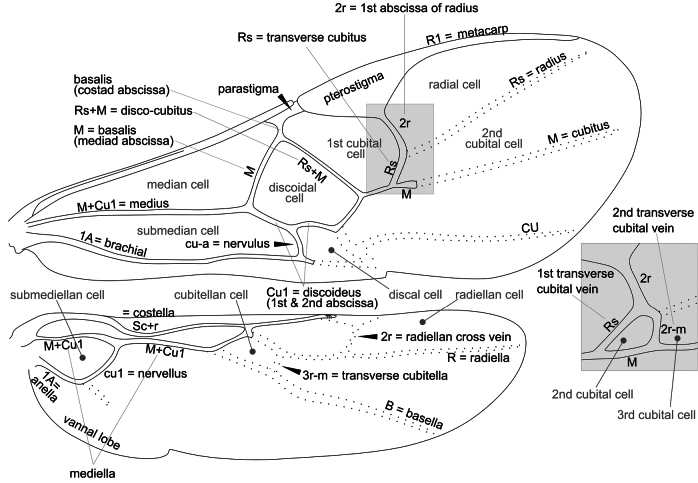
Wing venation and cells based on Nixon (1965) and deductions from his later publications. Wing vein terminology in black, wing cell terminology in grey. The inset shows a detail of the wing venation if a complete areolet is present.

**Figure 23.**
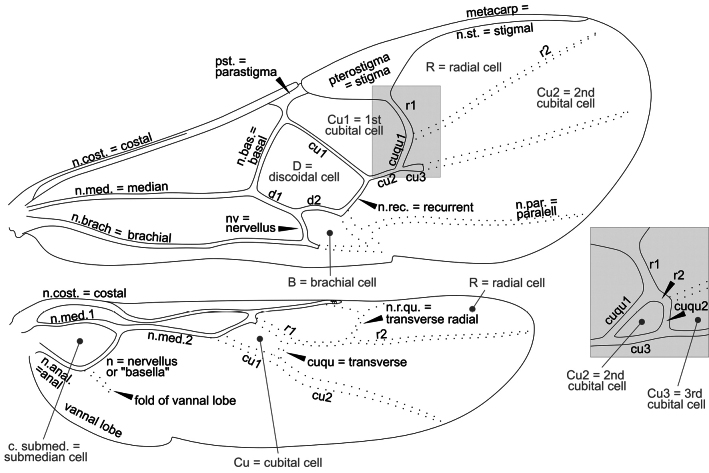
Wing venation and cells based on Papp (1976a, 1983). Wing vein terminology in black (“vein” omitted for simplicity e.g., costal vein = costal), wing cell terminology in grey. The inset shows a detail of the wing venation if a complete areolet is present. Terminology: fore wing: cu1, cu2, and cu3 = three sections of cubital vein; cuqu1, cuqu2 = first and second transverse cubital vein, d1, d2 = two sections of discoidal vein; r1, r2 = two sections of radial vein. Hind wing: n. med. 1, n. med. 2 = two sections of median vein; cu1, cu2 = two sections of cubital vein; r1, r2 = two sections of radial vein.

**Figure 24.**
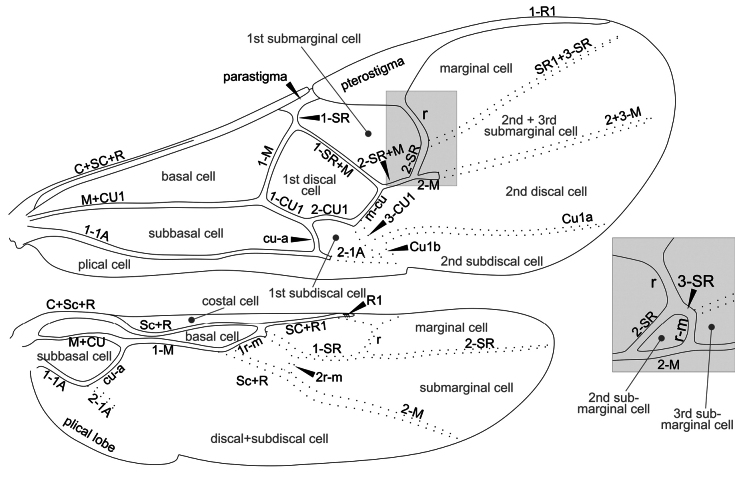
Wing venation and cells based on van Achterberg (1979). Wing vein terminology in black, wing cell terminology in grey. The inset shows a detail of the wing venation if a complete areolet is present. Terminology: veins: A = analis, C = costa, CU, cubitus, M = media, R = radius, SC = subcostal, SR = section radii, a = transverse anal vein, cu-a = transverse cubito-anal vein, m-cu = transverse medio-cubital vein, r = transverse radial vein, r-m = transverse radio-medial vein.

**Figure 25.**
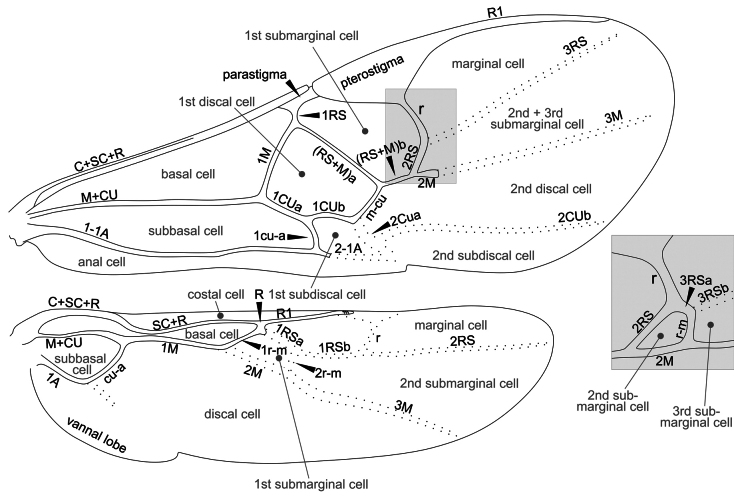
Wing venation and cells based on Sharkey and Wharton (1997). Wing vein terminology in black, wing cell terminology in grey. The inset shows a detail of the wing venation if a complete areolet is present. Based on Sharkey and Wharton (1997).

In the notes on genera below, we suggest the most useful sources for determination to species in each case. For now, at least, the only practical morphological approach to species-level identification of taxa with the areolet open (*Apanteles* s. l., in the old sense) will first involve Nixon’s and Papp’s keys. Although Papp (1988) attempted to list the European species of the old “*Apanteles*” into the genera employed by Mason (1981), his results were by no means always accepted (cf. Fernandez-Triana et al. 2020). For these reasons, one helpful prelude might be to annotate the full set of Nixon’s and Papp’s keys with current generic placements according to Fernandez-Triana et al. (2020). This will also reveal the (considerable) species-level synonymy that has taken place since the keys were produced, which is of course important when making and using species-level identifications. Access to Nixon’s and Papp’s keys is best sought through their keys to species groups (Nixon 1973 and Papp 1976a, respectively). It is important to appreciate that, although Nixon’s work is easier to use it is limited to NW Europe, and Papp’s includes many additional species and also proposes considerable synonymy relegating names employed by Nixon.

**Figure 26.**
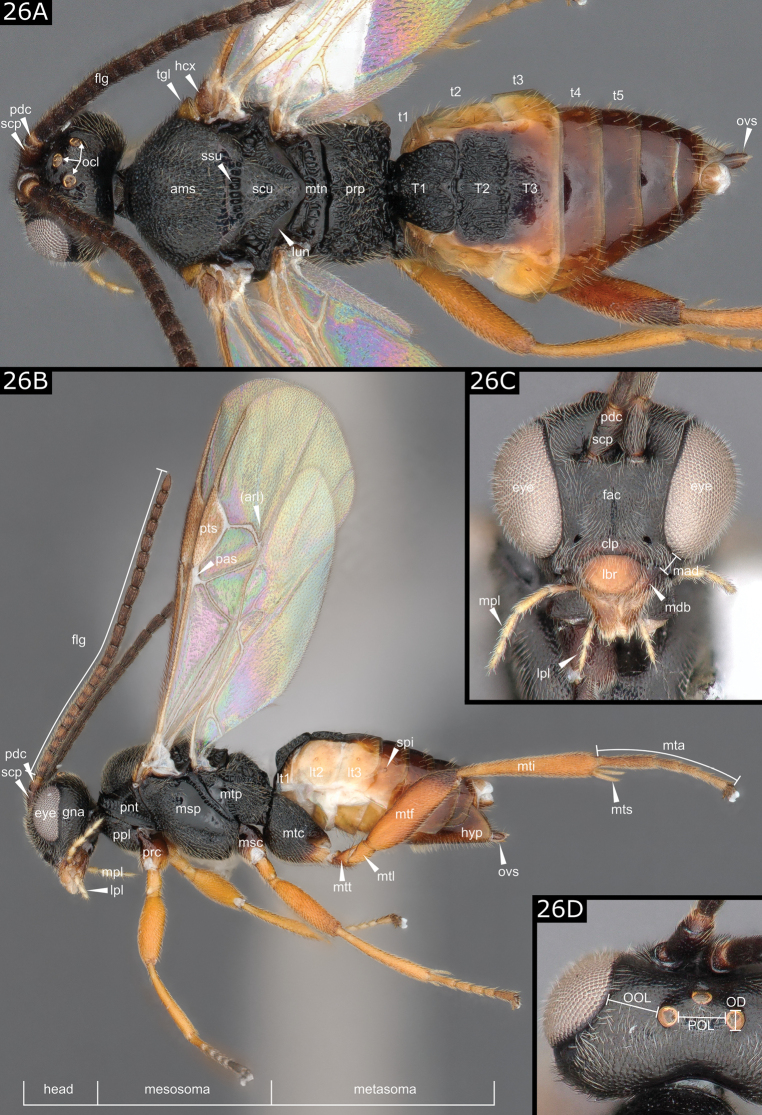
**A, B** (*Cotesia* sp.) **C, D** (*Illidopssplendidus*). Terminology following HAO (http://portal.hymao.org/projects/32/public/ontology/), historical terms between parentheses. Abbreviations: ams = anteromesoscutum (mesoscutum), clp = clypeus, eye = compound eye, fac = face, flg = flagellomere, gna = gena (temple), hcx = humeral complex, lbr = labrum, lpl = labial palpus, lt1–lt3 = laterotergites 1–3, lun = lunula, mad = malar distance (malar space), mdb = mandible, mpl = maxillary palpus, msp = mesopleura, mta = metatarsus (hind tarsus), mtc = metacoxa (hind coxa), mtf = metafemur (hind femur), mti = metatibia (hind tibia), mtl = metatrochantellus (hind trochantellus), mtn = metanotum, mtp = metapleura, mts = metatibial spurs, mtt = metatrochanter (hind trochanter), ocl = ocelli, OOL = distance between compound eye and a posterior ocellus, OD = diameter of a posterior ocellus, POL = distance between posterior ocelli, ovs = ovipositor sheaths, pas = parastigma, pdc = pedicel, pnt = pronotum, pts = pterostigma (stigma), prc = procoxa (front coxa), prp = propodeum, ppl = propleuron, scp = scape, scu = scutellar disc (scutellum), spi = spiracle, ssu = scutoscutellar sulcus (scutellar sulcus), T1–T3 = mediotergites 1–3 (T2 has also been referred to as “basal field”), t1–t5 = tergites 1–5, tgl = tegula.

### ﻿Comments on the genera of Microgastrinae known from Europe (in alphabetical order)

The following notes apply only to the European fauna. In particular, the relative species richness of genera in Europe is not paralleled in the World fauna (see Fernandez-Triana et al. 2020) and, in all of the moderate- and large-sized genera, it is clear (especially from DNA studies) that even in Europe considerable numbers of species still await description. Among all European areas, the Iberian Peninsula remains the least well studied.

***Apanteles*.** Although this is one of the two most species-rich genera of Microgastrinae worldwide, in Europe it is not as speciose, with only 33 recorded species so far. Many species can be identified by using the keys by Nixon (the *lacteus* and *ater* groups of Nixon (1976), and parts of the *metacarpalis* group of Nixon (1973) for species with shorter ovipositors) and Papp (1980, *obscurus* and *ater* groups; 1981, *lacteus* group; 1984a, parts of *metacarpalis* group); there has been no meaningful advance on these works for the region, and the disjointed treatment by the above authors makes an overview approach to the genus particularly challenging. There are several species that are difficult to place between *Apanteles* and *Dolichogenidea*, the differentiation of which is based mainly on the shape and degree of setosity of the hind wing vannal lobe. There are also several species with short ovipositors in which the pleats of the hypopygium are sometimes not strong, which also presents difficulties in relation to *Pholetesor*. Species with biological data comprise mainly solitary parasitoids of concealed microlepidoptera hosts, but *A.sodalis* (Haliday) is gregarious.

***Choeras*.** There are 12 described species in Europe. The genus is treated by Nixon (1973) and Papp (1983) as the *parasitellae* group plus (Nixon 1973, Papp 1982) as the *validus* group, but not all species are included. A more recent revision (van Achterberg 2003) allows for the identification of ten of the European species but a more complete key to all Western Palaearctic species is given by Abdoli et al. (2019). Largely because of the variable extent of both the closure and nature of the areolet and of the sculpture on the propodeum, this genus is difficult to key, although several species are easily placeable from the distinctly wedge-shaped T1, short T2, and the characteristic appearance of the venation around the areolet (either closed, or with vein r rather straight and meeting the relatively long and straight vein 2RS at a clean angle marked with the stub of vein 3RS, but note that in *C.gielisi* the closure of the areolet can be almost vanishingly proximal and easily overlooked (Shaw 2021); to a lesser extent this also occurs in *C.validus*). Where known, the species are solitary parasitoids of concealed hosts; mainly moderately large ‘microlepidoptera’, with at least two species on Psychidae.

***Cotesia*.** One of the two most speciose genera in Europe with 106 species recorded from the region. This is also one of the most commonly collected genera, being especially frequently reared from caterpillars. Works by Nixon (1974, as the *glomeratus* group) and more extensively Papp (1986a, 1987, 1990 as the *glomeratus* group) allow for the identification of many species, but a significant number of additional species have been described or characterised since (e.g., Oltra and Michelena 1989; Oltra et al. 1996; Shaw 2003, 2007, 2009, 2022; Ruohomäki et al. 2013; Fujie et al. 2021; Shaw and Fernandez-Triana 2020; Shaw and Colom 2023). Although *Cotesia* is comparatively well-defined, both morphologically and genetically, in a few cases it is difficult to recognise when species have reduced sculpture (on the propodeum, T1, and T2), or when the shape of T1 and/or T2 resembles that of *Protapanteles* (e.g., *Cotesiachares* (Nixon), *C.glabrata*, *C.hispanica*). A few species have a more or less prominent spine on the apical segment of the protarsus, which is found in at least some species of several genera (best known in several *Protapanteles* and *Glyptapanteles*, but also in *Distatrix*, *Venanides*, and in *Apantelessodalis*). The genus comprises solitary and gregarious parasitoids of mostly, but not exclusively, exposed ‘macrolepidoptera’. Species are largely haemolymph feeders and, as happens in several other haemolymph-feeding genera such as *Microplitis*, the host often remains alive (but does not resume feeding) after eruption of the parasitoid larva(e). Several species are known to have succeeding generations on a single host generation, sometimes with large corresponding variation in brood sizes (but the habit also occurs in a few solitary species).

***Deuterixys*.** Only three species are currently recorded from Europe. The genus is characterised by the contrast between a mostly smooth and shiny propodeum (where only a strong median carina is clearly marked) with the mostly sculptured T1 (at least on posterior half), T2, and usually T3 (partially or even entirely), and the notched appearance of the metasoma at the T2/T3 boundary. Species can be identified using the illustrations figured here (Fig. 19A–C); they are keyed by Papp (1983), Zeng et al. (2011), and Zheng and Song (2020). They are all solitary parasitoids of Bucculatricidae, emerging as adults from the host cocoon, and rather small.

***Diolcogaster*.** At present there are 14 species recorded for the region; a small representation of this largely tropical and morphologically diverse genus. There is no single key to species, but Nixon (1965) covers them in different groupings under his concept of *Protomicroplitis* Ashmead and, although laboriously, most European species can be determined through his keys. Some, including *D.mayae* (Shestakov) which is not treated by Nixon (1965), are illustrated by Ghafouri Moghaddam et al. (2019). Notes on the biology of three species, including *D.procris* (Fischer) that was not included by Nixon (1965), are given by Shaw (2012). Morphologically *Diolcogaster* has rather distinctive characters, although with great diversity in appearance, with *Rasivalva* being the only genus it could be confused with, particularly in species with reduced sculpture. Solitary or gregarious parasitoids, largely of ‘macrolepidoptera’.

***Distatrix***. A small genus with three species recorded from Europe. Nixon (1965) included them in his key to the *Apantelesformosus* group, but only two are included in his 1973 key; Papp (1984a, *formosus* group) keyed all three. In most species T1 is paler in colour than the posterior part of the metasoma; the lack of setae on the ovipositor sheaths is an important diagnostic character. Solitary or gregarious parasitoids of ‘macrolepidoptera’.

***Dolichogenidea*.** One of the two most speciose genera in Europe with at least 109 species recorded from the region, and one of the most commonly collected genera. Works by Nixon (1972, *laevigatus* group; 1973, most of *metacarpalis* group; 1976, *ultor* group) and Papp (1978, 1979, both *laevigatus* group; 1980, *lineipes* group; 1981, *longipalpis* and *ultor* groups; 1984a, parts of *metacarpalis* group) allow for the identification of some species, but the keys (and especially their arrangements) are not entirely satisfactory. At least part of the genus (the *laevigatus* group of Nixon) is relatively distinctive morphologically, based on length of ovipositor sheaths, pleated hypopygium and wing structure and venation. The shape and setae of the hind wing vannal lobe is the main character to separate it from *Apanteles*; however, some *Dolichogenidea* species such as *D.sicaria* have a more or less straight vannal lobe without setae and could be confused with *Apanteles*. All species recorded from Europe are solitary parasitoids as far as is known, and ectophagy in the final instar has been observed in several species. All *Dolichogenidea* parasitise at least weakly concealed hosts, including those that start life as leaf-miners (e.g., Coleophoridae); in those cases, some species have ovipositor sheaths as short as in *Pholetesor* (parasitising largely Gracillariidae, Elachistidae and in at least one case Bucculatricidae), and the distinction between the two genera is then difficult, especially as the hypopygium pleats are sometimes weak and inconspicuous when the ovipositor is short.

***Glyptapanteles*.** A moderately large and somewhat disparate genus, with 35 species recorded from Europe. Works by Nixon (1973, most of *vitripennis*, all of *pallipes*, *octonarius*, and *fraternus* groups) and Papp (1983, most of *vitripennis*, all of *fraternus*, *liparidis*, *octonarius*, and *thompsoni* groups) allow for the identification of most species, albeit without clear overview of the genus. Some recent additions for the area are illustrated by Höcherl et al. (2024). Some species of *Protapanteles*, e.g., *P.anchisiades* (Nixon), are morphologically close to several *Glyptapanteles* species. *Glyptapantelesmoldavicus* (Tobias), comb. nov. parasitises Bucculatricidae, and has at times been placed within either *Venanides* or *Pholetesor* but has also been considered to represent a very abnormal *Glyptapanteles* (see further comments in Fernandez-Triana et al. 2020). Here we formally transfer it to *Glyptapanteles* and the species will key to that genus in our key above. *Glyptapanteles* includes solitary and gregarious parasitoids, mostly of ‘macrolepidoptera’. A few species (e.g., *G.vitripennis* (Curtis)) are extremely polyphagous, though in other apparent cases (e.g., *G.fulvipes* (Haliday)) species complexes are probably involved.

***Hygroplitis*.** A small genus of relatively large species, sometimes treated as a species group within *Microgaster*. Three species are recorded from Europe, keyed by Shaw (1992). The hypopygium varies from practically fully sclerotised to clearly pleated. Solitary parasitoids of moderately large concealed ‘microlepidoptera’ and associated with wetlands, but poorly documented biologically.

***Iconella*.** There are 11 described species in Europe. Works by Nixon (1976, *merula* group) and Papp (1982, *merula* group, most of *laspeyresiella* group [= *mycetophilus* group sensu Papp 1976a], but this includes species now (Fernandez-Triana et al. 2020) regarded as both *Apanteles* s. str. (*A.nephus* Papp) and *Dolichogenidea* (*laspeyresiella* (Papp)) itself), allow for the identification of some species, but more recent papers (Kotenko 2007b and references cited therein) should also be consulted. The strong median carina and relatively large scutellar lunulae are diagnostic features. A small genus of solitary parasitoids of concealed ‘microlepidoptera’.

***Illidops*.** There are 13 described species in Europe. There is no satisfactory revision of this genus that allows for the identification of its species; Nixon (1976, *butalidis* group) deals with only a few species, rather unsuccessfully, and Papp (1981, *butalidis* group; 1984a, *suevus* group), although dealing with more species, only partly resolves this. Kotenko (2007a) treats five species from Europe and includes more species from further East. We (as Nixon did) have found that limits of species are sometimes very difficult to define (at generic level, too, the characters given in the key do not always work well because some species lack one or several of them). A small genus of solitary parasitoids of concealed ‘microlepidoptera’ (e.g., Scythrididae, and in one case epichnopterigine Psychidae).

***Microgaster*.** There are 54 described species in Europe. For a period between 1982 and 1988 this genus was sometimes known as *Lissogaster* (e.g., Tobias and Kotenko 1986). A moderately large genus, with mostly quite large species. Nixon (1968) and Papp (1976b) allow for the identification of many species, but recent papers (Shaw 2004, 2012, 2023a) should also be consulted. The mesoscutum is usually strongly shiny at least posteriorly, regardless of whatever sculpture may be present; and the hypopygium is usually pleated, but sometimes (e.g., *M.raschkiellae* Shaw) weakly so and in *M.meridiana* Haliday and *M.acilia* Nixon only creased. Mostly solitary (but *M.subcompleta* Nees is gregarious) parasitoids of weakly concealed hosts, typically ‘microlepidoptera’ but including some specialists on ‘macrolepidoptera’. The final instar larva is ectophagous (illustrated by Shaw 2004), explaining the exclusive connection with hosts resting in a concealed site.

***Microplitis*.** There are 62 described species in Europe. For a period between 1982 and 1988 this genus was sometimes known as *Microgaster* (e.g., Papp 1984b, 1986b; Tobias and Kotenko 1986). A moderately large genus. Nixon (1970), Papp (1984b) and Kotenko (2007a) allow for the identification of many species, but this remains a difficult and poorly resolved group, in part due to relatively large intraspecific morphological variability. Some species newly recorded in Europe are illustrated by Höcherl et al. (2024). The ovipositor often scarcely projects beyond the hypopygium (but, in rare cases, it can appear to do so because the hypopygium is greatly extended and so narrowed posteriorly that it becomes inconspicuous, e.g., *M.impressus* (Wesmael): cf. Shaw 2012); the hypopygium is fully sclerotised and usually has the margin acute, rarely truncate or emarginate (*M.ocellatae* (Bouché)); the head and mesosoma usually have rather characteristic matt/granulose sculpture (although there are lustrous species in eremic areas; e.g., Papp 1986b; Papp and Shaw 2001). The first metasomal tergite varies greatly in both shape and sculpture but the second tergite is usually only weakly sculptured. Solitary or gregarious parasitoids, almost entirely of exposed larvae of ‘macrolepidoptera’. Haemolymph feeders, so the host often lives beyond the eruption of the parasitoid(s) but does not resume feeding.

***Napamus*.** Only one European species, treated by Nixon (1965, 1973, 1976) as the *Apantelesvipio* group, and more recently by Ghafouri Moghaddam et al. (2021), known as a solitary parasitoid of concealed ‘microlepidoptera’. Beside the characters given in the key above, the galea (a lobe in the mouthparts) is conspicuously lengthened, ~ 2 × as long as wide.

***Paroplitis*.** There are two described species in Europe; the only common one (treated by Nixon (1965) as the monobasic *wesmaeli* group of *Hypomicrogaster*, and by Fujie et al. (2021)) is a gregarious parasitoid of scopariine Crambidae feeding in mosses (Shaw 2012). *Paroplitis* species have a very small areolet, its unpigmented outer side sometimes difficult to appreciate, resulting in some specimens appearing not to have an areolet. Propodeum with median longitudinal keel for at least part of its length.

***Pholetesor*.** (See also notes under *Dolichogenidea*.) There are approximately 15 species recognised in Europe, though with sometimes unclear species boundaries and some nomenclatural confusion (cf. Shaw 2012). Nixon (1973, *circumscriptus group*) and Papp (1983, *circumscriptus* group) key most European species. In death, the hypopygium appearing unusually strongly angled with the sternite anterior to it (= metasomal sternite 5) is a distinctive feature of several common species (Fig. 8B). Solitary parasitoids of Gracillariidae, Elachistidae, Bucculatricidae, Tischeriidae, and possibly other leaf-miners; some species sling their characteristic cocoons hammock-like across space created by the host and are frequently reared from Gracillariidae in particular.

***Protapanteles*.** A rather small, perhaps poorly justified (e.g., Fernandez-Triana et al. 2020), genus of parasitoids of ‘macrolepidoptera’ with ~ 15 described European species; most are solitary but a few are gregarious. Nixon (1976, *popularis* group) and 1973 (*triangulator* group) covers most species, although including *Cotesiachares* (Nixon) and omitting *P.anchisiades* which he treats as a species of his *vitripennis* group (i.e., *Glyptapanteles*). Papp (1984a, *popularis* group) provides more accurate coverage. A more recent species was described by Oltra et al. (1995). As currently constituted, *Protapanteles* contains some very disparate elements (e.g., *P.anchisiades*, *P.parallelus*, *P.santolinae*, *P.triangulator*). Also, some species of *Cotesia* with smooth propodeum, T1 and T2 look very similar to some *Protapanteles* and it can be difficult to decide their generic placement.

***Rasivalva*.** The key by Oltra-Moscardó and Jiménez-Peydró (2005) serves as a basis to identify the six described European species (see also Papp 1989). The statement in Fernandez-Triana et al. (2020) that the species *Rasivalvaleleji* Kotenko occurs in Ukraine was an error and should be disregarded; Kotenko (2007a) only mentioned that species as present in the Russian Far East, and there is no evidence whatsoever to support its presence in Europe. A smaller but similar group to *Diolcogaster* (and similarly highly diverse in appearance), but biologically less well known, although two species are solitary parasitoids of exposed Geometridae and a third of lithosiin Arctiinae (Erebidae).

***Sathon*.** Three sometimes common species in Europe. The genus was revised at a global scale by Williams (1988). *Sathonfalcatus* (Nees), with its long downcurved ovipositor and remarkably large male genitalia, was treated by Nixon (1965, 1973) as the only European representative of his *falcatus* group. It is a familiar grassland species, known as a gregarious parasitoid of the noctuid *Apameamonoglypha* (Hufnagel) making characteristic honeycomb-like cocoon batches, while the two European species of the *lateralis* species group (included by Nixon, 1973 in his *vitripennis* group), with their shorter and nearly straight ovipositors, are solitary parasitoids of Choreutidae. It is questionable how closely related these seemingly disparate elements are, and how this genus will be treated in the future (e.g., Fernandez-Triana et al. 2020).

***Venanides*.** The only European species is a solitary parasitoid of a species of Chimbachidae (Shaw 2020), but elsewhere the genus includes gregarious parasitoids.

## ﻿Additional information

### Author ORCIDs

Mark R. Shaw https://orcid.org/0000-0002-6651-8801

Amelie Höcherl https://orcid.org/0009-0007-4211-7468

Jose Fernandez-Triana https://orcid.org/0000-0003-0425-0309

### Data availability

All of the data that support the findings of this study are available in the main text.
